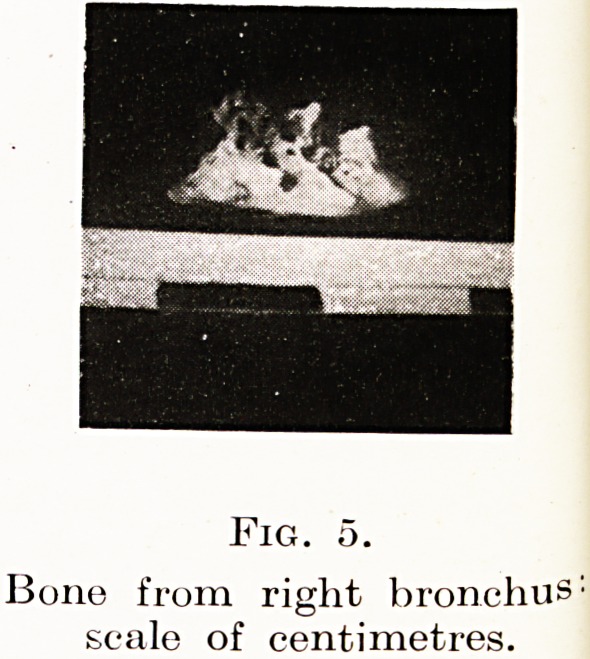# "Look and See," Foreign Bodies in Air and Food Passages

**Published:** 1944

**Authors:** E. Watson-Williams

**Affiliations:** Surgeon-in-Charge, Ear, Nose and Throat Department, Bristol Royal Hospital; Clinical Lecturer in Diseases of the Ear, Nose and Throat, University of Bristol; Laryngologist, Bristol National Radium Centre


### "LOOK AND SEE,"

### Foreign Bodies in Air and Food Passages,

BY

E. Watson-Williams, M.C., Ch.M., F.R.C.S.

*Surgeon-in-Charge, Ear, Nose and Throat Department, Bristol Royal Hospital ; Clinical Lecturer in Diseases of the Ear, Nose and Throat, University of Bristol ;*
*Laryngologist, Bristol National Radium Centre.*

---

When a patient complains of "something stuck in the throat," there is only one sound rule : examination *by endoscopy* as soon as arrangements can be made. We may make an exception when the object in question is known to be metallic, for even a small fragment of metal shows readily in a radiographic film. But it is extremely difficult to detect small portions of bone within the thorax, amid the crossing shadows of ribs and vertebral processes, while cartilage, glass, "composition" buttons, and so forth, may be completely transradiant.

It is not always easy to keep this rule. One may examine five or six patients in succession with negative results ; yet the seventh may be in actual danger from such an intruder, though presenting exactly similar symptoms. Insane persons, convicts and those awaiting trial not uncommonly swallow pins, nails, etc., to earn removal to hospital for investigation. Fortunately such persons generally choose metallic bodies. Sometimes also one encounters a patient who repeatedly claims to have something stuck in the throat. Quite recently I saw a young woman who made this claim three times in eight months, without any foundation, but appearing genuinely distressed each time. The only apparent motive was to get her husband home on compassionate leave !

The case may present as one of disease of these parts with no history pointing to the presence of a foreign body. The patient may be actually ignorant of any accident, too young or too frightened to tell about it—or even disbelieved when giving a clear and definite history.

Finally, the fact that a foreign body has been coughed up or has passed *per vias naturales* is not conclusive : there may be more than one.

Endoscopy is a simple and safe procedure. The worst treatment possible is blind poking about with a probang or coin-catcher—ingenious devices of the last century whose only place is now in the Museum.

**Case 1.** Anita O., aged 7, was admitted in 1931, with severe respiratory obstruction—so severe that all the accessory muscles were called into action and there was very pronounced " sucking-in " not only of ribs and lung apices, but also at the sides of the trachea : this suggesting that the obstruction was above the sternum. The history was given that the condition had come on during six weeks, since the child had choked over an orange pip. The voice was weak, but clear : coughing very feeble, no expectoration : slight evening pyrexia.

Under local (" Tutocaine ") anæsthesia I carried out direct laryngoscopy. No foreign body seen, glottis normal : the lumen of the trachea appeared to be completely obstructed by granulations. A soft rubber tube was passed through this mass, giving immediate relief ; and a general anæsthetic administered. Tracheotomy was then performed, dividing the second, third and fourth rings of the trachea. No foreign body was found, so the " granulations " were removed as thoroughly as possible and the tracheotomy tube left in situ. The material removed was reported " round-celled sarcoma." I inserted radium 25 milligrammes, filter 0.5 mm. platinum and 0.5 mm. gold, total dose 3 gramme-hours : respiration was carried on through a *lower* tracheotomy.

In removing the radium, after the anchoring stitches had been removed, the safety silk broke, and the container fell down the trachea blocking it completely. The child was seized by the pelvis, head-down : a good shake shot the container into sight, and it was removed. As I mopped my forehead she whispered, " I didn't cry, did I, Sister ? "

The child made a complete recovery, although the tracheal cartilages perished after the irradiation and the tube had to be retained. She became head of her school, was captain of tennis, gymnastics and hockey (and felt sure she could have been captain of swimming, had it not been for the tracheotomy !) and won a county scholarship. I have heard recently that she is married and has a child, born in 1943. It is curious that her father died of cancer of the bronchus in 1937.

**Case 2.** Nine years ago a large, robust butcher, aged 48, came to me complaining of gradually increasing difficulty in swallowing during six months. No solids could pass, and even fluids were sometimes returned : it felt as if the obstruction was behind the lower end of the sternum. There was occasional dull, aching pain in the back behind that point. He had lost a stone in weight, but was certainly not thin. X-rays showed a stricture of the œsophagus 2 inches above the diaphragm, with moderate dilatation above. Diagnosis : cancer of œsophagus.

I passed an œsophagoscope, and 34 cm. from the incisor teeth. I found a green foreign body, rather firmly held by spasm of the œsophageal walls, which were ulcerated. On removal it proved to be a flake of bone, roughly circular and 8 mm. in diameter. He had no recollection of swallowing it ; but had been in the habit of

FIG. 1.

Hair-pins in œsophageal entrance.

FIG. 2.
The hair-pins after removal: scale of centimetres.

Fig. 3.
Right Bronchiectasis: Note that right side of chest appears on right.

Fig. 4.
Lateral view of bronchiectasis.

Fig. 5.
Bone from right bronchus: scale of centimetres.

eating scraps of raw fat while at work and thought it might have been swallowed accidentally in this way. It is unusual for a foreign body to become arrested so far down in the œsophagus. Recovery was rapid and complete.

**Case 3.** Miss E., 49, was sent to me in 1942, complaining of slight discomfort in the region of the right tonsil, and inability to swallow hard solids for some years. She had had this difficulty with hard solids all her life. Four years previously she was depressed, and it was necessary to certify her. On arrival at the mental home she realized what was happening, and when alone for a moment rolled up two hair-pins into a ball and swallowed them. At once she became inarticulate, but pointed to her throat and made it clear that she thought there was something stuck there; but this symptom had been present for some time, and was not specially regarded. She soon regained her power of speech, but could—or as it was considered, would—take nothing but slops. She complained several times of attacks of severe pain high up in the throat, or at the back of the mouth; and told her attendants that she had swallowed hair-pins. But she was assured that these had passed per vias naturales, and urged not to let herself dwell on the delusion, which was the only remaining unfavourable mental symptom.

As her general mental condition had improved she was discharged to the care of relatives; and although she continued to complain of the hair-pins in the throat, her friends and doctor urged her to forget it (in fact, it seems that she was told if she continued to complain she would have to return to the Mental Hospital).

In July, 1942, she consulted another doctor, who felt that "to satisfy her" an X-ray examination should be made; upon which a tangled mass of wire was shown in the region of the epiglottis (Figures 1, 2). When she came to me it was easy to see loops of metal behind the base of the tongue, but the mass was so firmly embedded that to remove it a general anæsthetic and wire cutters were found necessary.

**Case 4.** Mr. L., 50, was sent to me recently with the diagnosis of cancer of the bronchus. In October, 1943, he began to be troubled with shortness of breath, wheeziness and expectoration—the latter rapidly became profuse and offensive. Three weeks before this he had choked over a bone, he said, while eating mutton. But there was no difficulty in swallowing, and as his own doctor was ill he thought little of it; though he mentioned the incident when he saw the doctor for the "bronchitis." He was sent to the local hospital and his name was put down for admission, but after five months, as symptoms increased and he was not sent for, I was asked to see him.

An X-ray showed considerable opacity in the right lower lobe; and the fetid sputum was obviously coming from a bronchiectasis or similar lesion. None of the X-rays showed any foreign body (Figures 3, 4), so the history of this had been considered unimportant.

On passing a bronchoscope I found the right main bronchus full of foul pus, which welled up freely: so that several minutes had to be devoted to removing this by suction before proceeding further. The walls of the bronchus were sodden and inflamed and at the junction of the eparterial bronchus I found a white irregular bone embedded in granulations. It was so large (Figure 5) that it would not pass through a 10 mm. bronchoscope and therefore had to be withdrawn in the forceps protruding from the end of the instrument. Unfortunately severe damage has been done to the lung by this protracted stay and the ultimate prognosis is not good.

**Case 5.** A mongol idiot, aged 3, was admitted to a local hospital with acute intestinal obstruction. The condition resolved when an enema brought away a large "composition" coat-button. But ten days later the child was refusing all food, though it would drink. The mother was sure there must be another button stuck in the throat. X-rays showed no foreign body; though when barium was given there was some delay in passing this from the level of the upper edge of the sternum.

Under ethyl chloride anæsthesia I passed a laryngoscope and found a button of 28 mm. diameter (a little larger than a half-penny), rather firmly gripped in the œsophagus, the upper edge 1.5 cm. below the cricoid; the lateral walls of the œsophagus were ulcerated and bleeding. I was able to break the button up into five pieces and thus to remove it—making sure, of course, that the pieces fitted together to make the complete button.

These five cases illustrate the great importance of an early and thorough examination *by endoscopy* in all cases of disease of the œsophagus or the main bronchial passages, and particularly if there is even a possibility of a foreign body having lodged there. While it is rare for life to be immediately threatened, unless larynx or trachea is involved, there should be no delay in getting expert help; the complications that may follow retention of a foreign body in these parts are often crippling and sometimes fatal.

### SUMMARY.

The five cases cited may be summarized thus :—

1. Suspected foreign body in trachea found to be sarcoma.
2. Supposed cancer of œsophagus found to be unsuspected foreign body.
3. Foreign body in hypo-pharynx, for years dismissed as delusion.
4. Supposed cancer of bronchus found to be foreign body; severe bronchiectasis owing to delay in examination.
5. Second foreign body in œsophagus of idiot, first having passed per vias naturales.

## Figures and Tables

**Fig. 1. f1:**
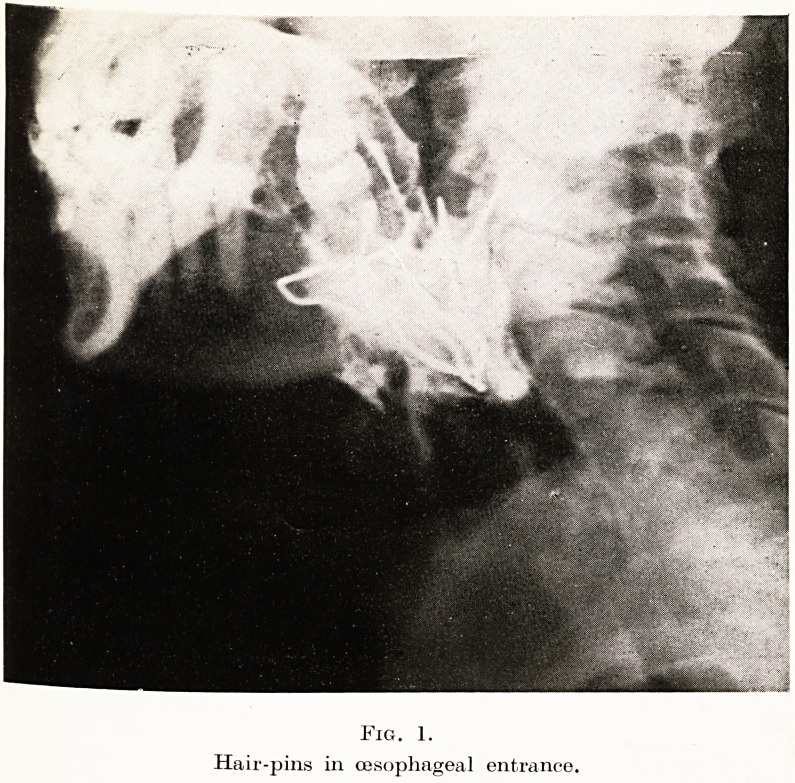


**Fig. 2. f2:**
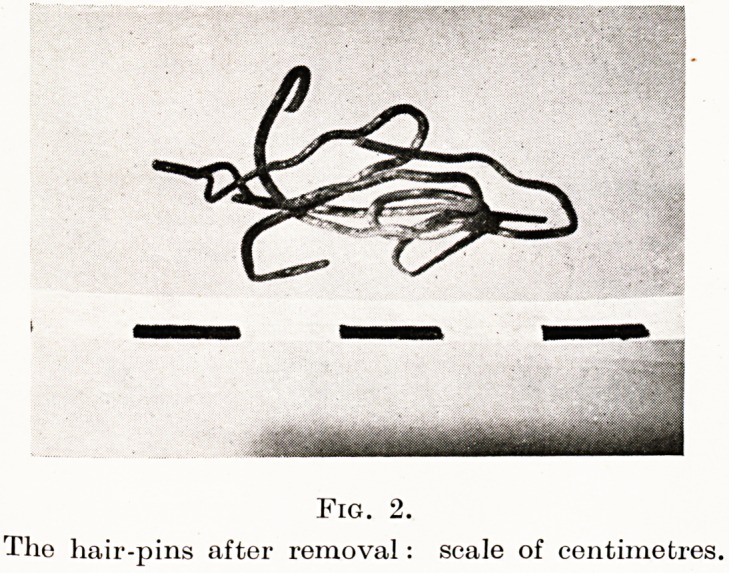


**Fig. 3. f3:**
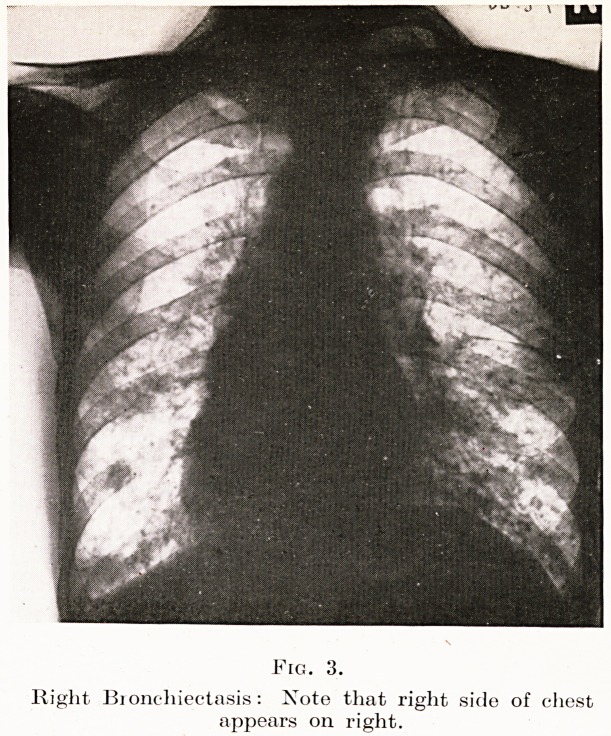


**Fig. 4. f4:**
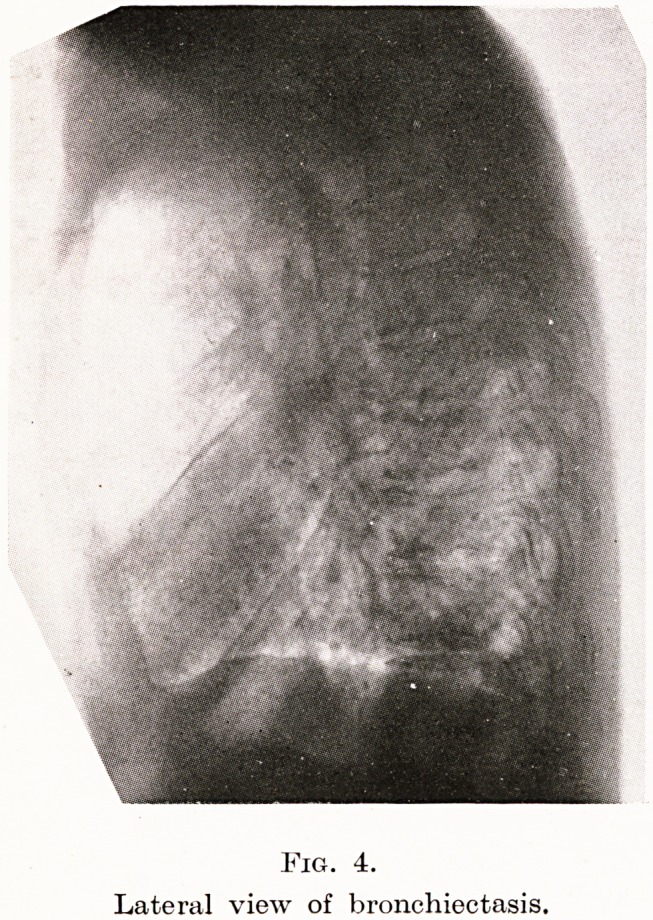


**Fig. 5. f5:**